# Effects of a Comprehensive, Intensive Lifestyle Intervention Combined with Metformin Extended Release in Obese Adolescents

**DOI:** 10.1155/2014/659410

**Published:** 2014-11-10

**Authors:** Cheril L. Clarson, Hilary K. Brown, Stefanie De Jesus, Michelle Jackman, Farid H. Mahmud, Harry Prapavessis, Tracy Robinson, J. Kevin Shoemaker, Margaret Watson, A. Justine Dowd, David J. Hill

**Affiliations:** ^1^Children's Hospital, London Health Sciences Centre, P.O. Box 5010, London, ON, Canada N6A 5W9; ^2^Lawson Health Research Institute, University of Western Ontario, London, ON, Canada N6A 5A5; ^3^Department of Paediatrics, University of Western Ontario, London, ON, Canada N6A 5A5; ^4^Department of Epidemiology & Biostatistics, University of Western Ontario, London, ON, Canada N6A 5A5; ^5^School of Kinesiology, University of Western Ontario, London, ON, Canada N6A 5A5; ^6^Department of Medicine, University of Western Ontario, London, ON, Canada N6A 5A5; ^7^Department of Physiology and Pharmacology, University of Western Ontario, London, ON, Canada N6A 5A5

## Abstract

*Objective*. To assess a comprehensive, intensive lifestyle intervention in combination with metformin extended release (MXR) or placebo on body mass index (BMI) and risk factors for type 2 diabetes and cardiovascular disease in obese adolescents. *Study Design*. Sixty-nineobese adolescents (mean BMI 32.5) received a comprehensive lifestyle intervention with structured dietary, physical activity, and behavioral components for 24 months. Subjects were randomized to 1 of 4 groups: MXR (33) 2,000 mg daily or placebo, with either moderate or vigorous intensity exercise for the first 3 months. Subsequently the exercise intervention was the same for all 4 groups. *Results.* Anthropometry measurements did not differ with initial exercise intensity at any time. At 3 months % body fat decreased in all 4 groups (*P* < 0.006). BMI and % body fat decreased in the MXR groups, but not the placebo groups, at 6 (−0.88, −3.16) and 12 months (−0.56, −2.34) (*P* < 0.05). Insulin resistance, fasting blood glucose, and leptin improved in all groups at 6 and 12 months. A high subject attrition rate (58%) occurred by 24 months. *Conclusion*. A comprehensive, intensive lifestyle intervention combined with MXR led to a decline in BMI and % body fat at 1 year independent of initial exercise intensity. This trial is registered with ClinicalTrials.gov NCT00934570 .

## 1. Background

The prevalence of childhood obesity in Canada, as in other developed countries, has increased dramatically. In 1978, 15% of Canadian children were overweight or obese, but this had risen to 31.5% in 2011 [1, 2]. Not only is childhood obesity a strong indicator of future obesity in adulthood, but also it confers a high degree of risk for progression to type 2 diabetes (T2D) and cardiovascular disease (CVD) [3, 4]. Ninety-five percent of Canadian children with T2D are obese at diagnosis [5]. Although there are multiple risk factors for T2D, obesity has been identified as one of the most significant [6, 7].

The two major strategies for management of adolescent obesity and associated metabolic risk are lifestyle modification and pharmacologic therapy. Comprehensive lifestyle interventions, including diet, physical activity, and behavioral strategies, have demonstrated improvements in BMI and metabolic risk in obese adolescents, but most studies are limited to 6 months or less and long term sustainability has not been addressed [8–10]. Although physical activity is a fundamental component of lifestyle interventions, the type of activity which is most beneficial with respect to BMI and metabolic profile is not clear. There is conflicting evidence as to whether vigorous or high intensity interval training rather than moderate or standard activity is more effective in promoting improved BMI and associated metabolic parameters [11–13]. Parental/family involvement in delivering lifestyle interventions has been shown to contribute to improvement in BMI [14, 15]. With respect to pharmacologic therapy, metformin has been shown to be effective in reducing BMI in obese adolescents, with variable effects on metabolic parameters, in randomized controlled trials of six months duration [16–19]. Although findings from these studies demonstrated improvements in anthropometry with metformin, this was not combined with an individually structured lifestyle component, which is likely important to sustain improved BMI. We have reported previously that metformin in combination with a six-month structured lifestyle intervention was effective in reducing BMI in obese adolescents [20]. While many other studies combining lifestyle interventions with metformin have been shown to have a moderate positive impact on BMI, these have been limited to six to 12 months in duration [21–24]. Metformin is available in an extended release formulation permitting once daily dosing fostering compliance with reduced side effects [25]. There is a single published report of intervention with metformin extended release (MXR) in obese adolescents where a significant but small improvement in BMI *z*-score of −0.09 was found at 1 year [26]. This study did include a lifestyle intervention but this was of limited intensity after the first ten weeks.

We hypothesized that a longer duration, more intensive lifestyle intervention in combination with MXR pharmacologic therapy may be necessary to promote sustainable improvement in BMI. We also aimed to compare the impact of an initial moderate intensity versus vigorous intensity exercise program. The REACH (Activity and Metformin Intervention in Obese Adolescents) study was implemented as a randomized, double blind, placebo-controlled trial of MXR in combination with a comprehensive, intensive lifestyle intervention. The primary objective of the trial was to assess the effect of this intervention on obese adolescents, compared to an identical lifestyle intervention in combination with placebo. Secondary objectives were to evaluate changes in body composition % body fat and risk factors for T2D and CVD.

## 2. Methods

### 2.1. Participants

Potential participants aged 10 to 16 years with a BMI above the 95th percentile for age and gender were identified from referrals by pediatricians and family physicians, newspaper, and radio print advertisement. Inclusion and exclusion criteria are detailed in the complete study protocol, which has been described previously [27].

### 2.2. Randomization and Blinding

Randomization was completed via a computer-generated randomized numbers table. The REACH study coordinator was responsible for enrolling participants and group assignment. All study personnel were blinded to medication and exercise group assignment, with the exception of the Exercise and Health Psychology Lab (EHPL) exercise specialist who was aware of exercise group assignment.

### 2.3. Metformin Intervention

Subjects were randomized to receive MXR (Glumetza) or placebo tablets and instructed to start taking 1 tablet per day (MXR 500 mg or placebo) and increase by 500 mg/day every 7 days to a maximum tolerated dose of 2,000 mg/day, taken before the evening meal as a single daily dose. All medications were supplied by London Health Sciences Centre pharmacy. Medication was dispensed weekly for the first 5 weeks and then every 4 weeks for the duration of the study. Medication compliance was assessed by the study coordinator with pill counts at each of these time points.

### 2.4. Lifestyle Intervention (Figure 1)

Subjects within the MXR and placebo groups were further randomized to engage in a moderate or vigorous intensity exercise program for the first 12 weeks of the study. The moderate intensity condition involved exercise at 40–55% of participants' heart rate reserve, compared to 60–75% of heart rate reserve for the vigorous intensity condition. Heart rate reserve was calculated based on predicted heart rate max (220 minus age). Resting heart rate was determined during baseline testing after lying at rest for 10 minutes. Each exercise session consisted of a warm-up, aerobic exercises (using treadmills, steppers, rowers, and bikes), resistance training, and cool down. The exercise intensity was built up gradually over the 12-week program, starting at 5 minutes of elevated intensity and working up to 30 minutes per 60-minute exercise session. Exercise specialists led subjects through the gradual increases in exercise intensity. Exercise intensity was monitored through heart rate monitors. Data from the heart rate monitors were downloaded after every session. Subjects exercised in groups of 8 to 12 for 1 hour, 3 times a week for weeks 1–6; 1 hour, twice a week for weeks 7–9; and 1 hour, once a week for weeks 10–12, supervised by fitness specialists. The exercise sessions were progressively less frequent to encourage subjects to incorporate independent physical activity into their own daily schedules. The fitness specialists supervised all exercise sessions at the EHPL and performed the fitness testing. After completing the 12-week exercise program, subjects continued with weekly supervised group exercise (moderate and vigorous groups combined) at a Community Centre (YMCA) and received a 2-year membership to encourage independent physical activity.

For the first 12 weeks of the intervention, subjects engaged in weekly group behavior change sessions based on the group-mediated cognitive-behavioral intervention model [28, 29]. Subjects and families had individual visits with a social worker and dietitian once a month for the first year and every 3 months during the second year, as well as group family sessions every three months. During the first 3 months of the trial there were 27 supervised group exercise, 12 group behavior change, 2 dietitian, and 2 social work sessions. For the remainder of the first year there were 39 supervised group exercise, 10 dietitian, 10 social worker, and 3 group family sessions, for a total of 86 hours of direct contact. During the second 12 months of the trial there were 52 supervised group exercise, 4 dietitian, 4 social worker, and four group family sessions for a total of 64 hours of direct contact. Exclusion criteria following study entry were nonadherence to the intervention assessed as less than 70% for medication and less than 50% attendance at fitness, nutrition, and social work sessions combined.

### 2.5. Measures and Procedures

At the baseline visit subjects were fasting and endothelial function was measured by peripheral arterial tonometry (Endo-PAT, Itamar Medical, Caesarea, Israel) [30]. An automated algorithm was used to calculate the reactive hyperemia PAT index (RH-PAT) for each subject. An oral glucose tolerance test (OGTT) was completed with measurement of fasting insulin, A1C, TSH, transaminases (AST and ALT), creatinine, HDL and LDL cholesterol, triglycerides, adiponectin, and leptin. Insulin resistance was estimated by the homeostasis model assessment insulin resistance index (HOMA-IR) = fasting insulin (mU/L) × fasting glucose (mmol/L)/22.5).

Weight and height were measured with a digital scale and a Harpenden stadiometer. BMI and BMI *z*-scores (kg/m^2^) were calculated from the U.S. Centers for Disease Control and Prevention reference data [31]. Percentage over BMI was calculated as the percentage of children above the 50th percentile BMI for age and gender [32]. Physical examination, including pubertal staging (Tanner) and blood pressure (BP), was done. Age and sex-adjusted *z*-scores for BP were calculated using the National Health and Nutrition Examination Survey (NHANES III) as a reference population [33]. The % body fat was determined by whole body DEXA analysis (iDXA; General Electric-Lunar iDXA, Ames Medical iDEXA; Prodigy, enCORE 2007 software version 11.40.004, Waukesha, WI). All measures were repeated at 6, 12, and 24 months.

The study was approved by The University of Western Ontario Research Ethics Board and Health Canada, and informed consent was obtained from all study subjects. The study was conducted from May 2009 to May 2012 within a single academic centre with all subjects attending sites at the Children's Hospital, London Health Sciences Centre, EHPL at The University of Western Ontario, and the YMCA. All sessions were conducted between 4 and 8 p.m. to facilitate attendance by adolescents after school and parents after work.

### 2.6. Statistical Analysis

Differences among the four randomization groups at baseline were tested using ANOVA (for continuous variables) or Fisher's exact test (for categorical variables). There were no differences between the four groups (MXR plus vigorous exercise, MXR plus moderate exercise, placebo plus vigorous exercise, and placebo plus moderate exercise), in terms of any of the baseline demographic, anthropometric, or metabolic variables. Two models were used to analyze the impact of exercise (vigorous versus moderate) or metformin on anthropometric and metabolic outcomes at 13 weeks and 6, 12, and 24 months, both using an intention-to-treat approach to analysis. In model 1, the four treatment groups were compared independently for outcome changes with time and between groups. However, because no differences were found for any outcome with respect to exercise intensity, the moderate and vigorous intensity exercise groups were subsequently combined within model 2 and assessed as two study groups, namely, lifestyle intervention with MXR and lifestyle intervention with placebo. Mixed factor ANOVA was performed using SAS PROC GENMOD to test the significance of the interactions between treatment group and time [34]. Statistically significant interactions denoted a differential effect of study group on measures over time. Analyses with statistically significant interaction terms were followed up with post hoc tests comparing differences across time within the treatment groups. Significance values for all post hoc tests were adjusted for multiple comparisons using a Tukey correction. SAS 9.2 was used for all analyses [35].

## 3. Results

### 3.1. Participants

**Figure 2:**
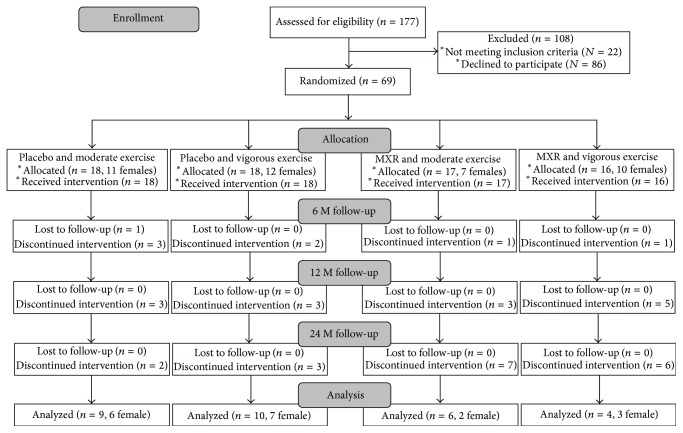
Flow chart for screening, enrollment, randomization, and follow-up of study participants.

One hundred and seventy-seven adolescents were screened and 69 were randomized: 33 (17 females) to the metformin group and 36 (23 females) to the placebo group (Figure 2). Within the metformin group 17 were randomized to initial moderate and 16 to initial vigorous intensity exercise and within the placebo group 18 were randomized to initial moderate and 18 to initial vigorous intensity exercise. Seven subjects were prepubertal at baseline, 26 were Tanner stage 2, 3, or 4, and 36 were Tanner stage 5. There were no statistically significant differences among the groups at baseline, with the exception of triglycerides (Table 1).

### 3.2. Loss to Follow-Up, Medication Adherence, and Session Attendance

Sixty-one subjects (88%) completed 6 months (34 females), 47 (68%) completed 12 months (27 females), and 29 (42%) completed 24 months (18 females) of study. There were no statistically significant differences between 24-month completers and noncompleters with respect to baseline characteristics, with the exception of triglycerides, which were significantly lower among completers than noncompleters (*t* = 2.10, *P* = 0.04). There were no differences in attrition between groups. Medication adherence was 84–87% at 6 months, 83–86% at 12 months, and 79–89% at 24 months, and medication compliance was not significantly different across time or between treatment groups. One subject was excluded prior to 6 months because of medication compliance of less than 70%. Attendance at fitness and nutrition/social work sessions decreased throughout the study in all four groups. Attendance at fitness sessions was 81–85% at 3 months, 57–76% at 3–6 months, 40–63% at 7–12 months, and 53–67% at 13–24 months. For nutrition and social work sessions attendance was 64–71% at 3–6 months, 53–79% at 7–12 months, and 50–72% at 13–24 months. There was no difference in the magnitude of the decrease between groups. Two subjects were excluded prior to 12 months because of attendance rates of less than 50%.

### 3.3. Model 1 Intention to Treat Analysis of Outcomes for Four Study Groups

At 13 weeks, there were no differential changes across time by treatment group for BMI, BMI *z*-score, % over BMI, or % body fat. Irrespective of treatment, intervention results showed no change in BMI or BMI *z*-score but a significant improvement in % body fat (*P* = 0.006) and % over BMI (*P* = 0.02) compared to baseline (Table 2).

At 6 months, there was no time by treatment group interaction effect for BMI, BMI *z*-score, or % body fat. An interaction effect, however, was found for % over BMI with a significant improvement between baseline and 6 months for MXR + vigorous (*P* < 0.002) and MXR + moderate (*P* < 0.005), but not for either placebo group (Table 2). At 6 months, there was also a differential change across time in HOMA-IR (*P* = 0.03) and adiponectin/leptin ratio (*P* = 0.04), depending on treatment group (Table 3). Specifically, a significant improvement in HOMA-IR occurred between baseline and 6 months for MXR + moderate (*P* = 0.005) and Placebo + vigorous (*P* < 0.001) but not for MXR + vigorous or Placebo + moderate which deteriorated (*P* < 0.02) (Table 3). A significant improvement in leptin was also seen between baseline and 6 months for MXR + vigorous (*P* < 0.02), MXR + moderate (*P* < 0.02), and Placebo + vigorous (*P* < 0.001), but not for Placebo + moderate (Table 3). There was a significant improvement in BMI *z*-score, % over BMI, % body fat, HOMA-IR, FBG, leptin, adiponectin/leptin ratio, and HDL cholesterol at six months compared with baseline overall, irrespective of exercise intensity or medication. However, cardiovascular parameters, systolic and diastolic BP and PAT values, were not altered (Table 3). Thus, after 6 months of intervention, there was evidence of a reduction in metabolic risk factors, but not cardiovascular disease risk factors, with those subjects receiving MXR showing the most significant changes.

By 12 months of intervention the significant improvements with time seen at 6 months in BMI, BMI *z*-score, % over BMI, % body fat, HOMA-IR, fasting glucose, leptin, and adiponectin/leptin ratio were maintained, but had not incrementally improved over the additional 6 month period (Table 2). Intervention-specific effects were found for % over BMI (*P* < 0.04) and HOMA-IR (*P* < 0.01) with the best individual group outcomes in the MXR + vigorous group. There were no further improvements in other BMI or metabolic measures between 12 and 24 months although interpretation of the 24 month data was limited by the small number of remaining subjects. The only observed change in a vascular parameter was a reduction in diastolic BP in the MXR + vigorous and MXR + moderate exercise groups. PAT-RH had deteriorated in treatment groups at 12 months compared with baseline. Equivalent results were found with log transformed PAT values (data not shown).

### 3.4. Model 2 Analysis of Outcomes by Combining the Exercise Intensity Groups

Treatment by time interaction effects was statistically significant for all the BMI outcomes as well as % body fat (Table 4). There was a differential change across time, depending on treatment group (MXR or placebo) for BMI, BMI *z*-score (*P* = 0.01), % over BMI (*P* = 0.01), and % body fat (*P* = 0.03). For BMI *z*-score, the MXR group showed a statistically significant decrease between baseline and 6 months (*P* < 0.0001) and baseline and 12 months (*P* < 0.0002). Percent body fat also decreased in the MXR group, between baseline and 6 months (*P* < 0.0001), baseline and 12 months (*P* = 0.0002), and baseline and 24 months (*P* = 0.03) (Table 2) There was no change in BMI, BMI *z*-score, or % body fat in the placebo group at any time point. There was no statistically significant differential change across time by treatment group for any other measure.

For the MXR and placebo groups in model 2, statistically significant changes occurred with time for HOMA-IR, FBG, leptin, and HDL cholesterol. For these outcomes, the pattern of change did not differ significantly between the two treatment groups. HOMA-IR decreased from baseline to 6 months (3.23 (2.22) to 2.19 (1.40), *P* = 0.03) and FBG also decreased at 6 months (4.88 (0.34) to 4.56 (0.35), *P* = 0.0002) but changes were not sustained at 12 and 24 months when values were unchanged from baseline. Leptin decreased at 6 (*P* = 0.003) and 12 (*P* = 0.02) months but subsequently increased between 12 and 24 months (*P* = 0.02). In the MXR group adiponectin and the adiponectin/leptin ratio were significantly greater than in the placebo group. HDL cholesterol increased between 6 and 24 months (*P* = 0.001), largely within the MXR group. When considering the MXR or placebo group there was no significant change in BP across time in either group, whilst PAT-RH had deteriorated in both treatment groups by 12 months.

### 3.5. Sensitivity Analyses

As a sensitivity analysis, BMI *z*-scores across the study period were assessed only among those with data at each time point (*n* = 29). There were no statistically significant differences among the groups at baseline. The mixed factor ANOVA analyses for completers showed similar results to those seen in main model 2 analysis, with statistically significant treatment group by time interaction effects. Follow-up tests for BMI *z*-score showed statistically significant differences in the MXR group only: between baseline and 6 months (*P* = 0.001) and baseline and 12 months (*P* = 0.009).

### 3.6. Safety

There were no severe medication related adverse events. There were two expected, nonserious events related to metformin: one subject had transient elevation of transaminases at one year which resolved one month after discontinuing the study drug and another subject discontinued the study drug for two weeks at one year due to persistent diarrhea. This was unchanged and the study drug was resumed. Six subjects were unable to tolerate the 2,000 mg dose of study drug and this was reduced to 1,500 mg in 4 subjects (2 MXR and 2 placebo) and to 1,000 mg in 2 subjects (both MXR).

## 4. Discussion

The comprehensive, intensive lifestyle intervention delivered in the REACH trial in combination with MXR resulted in a significant decrease in BMI *z*-score, % over BMI, and % body fat in obese adolescents. These improvements in anthropometric measures in the MXR group were evident at 6 months and sustained at 12 months. There were no differences between the MXR and control group in the magnitude of change across time for any other outcome measure, but both treatment groups showed similar improvements in metabolic parameters including HOMA-IR, FBG, and leptin. This highlights the value of lifestyle change as an essential part of an effective intervention.

The REACH lifestyle component alone was not associated with a decrease in BMI *z*-score or % body fat. This is notwithstanding significant effort to provide subjects with structured and unstructured exercise opportunities as well as positive reinforcement. Despite this, the lack of increase in BMI is positive since in the absence of intervention obese adolescents tend to have continued accelerated weight gain. Previously, in a six-month obesity intervention, we found an increase in BMI in adolescents managed with standard care comprising dietary and exercise advice only, and others have also reported that without active intervention obese adolescents continue to gain weight [20, 36, 37].

Since poor lifestyle is one of the most significant factors contributing to adolescent obesity and subsequent progression to T2D and CVD, identification of effective interventions leading to improved, sustained adolescent lifestyle is crucial [4, 6–8]. Components of lifestyle interventions found to impact positively BMI and metabolic risk include physical activity, nutrition education, behavioral therapy, and family involvement. The physical activity component of most adolescent obesity interventions has been of moderate intensity. We found that initial exercise intensity did not impact BMI or metabolic risk. This is consistent with a 12-week intervention in obese children aged between 8 and 12 years where endurance training and high intensity interval training were equally effective in improving BMI and insulin resistance [11]. A study in adolescent females also found no difference in the improvement in BMI and percentage body fat with high intensity interval training compared to moderate intensity interval training. In this study insulin resistance and dyslipidemia only improved in the high intensity group [38]. Others have reported that high intensity exercise training is superior to low intensity aerobic training in improving BMI, abdominal obesity, and cardiovascular markers [13, 39]. In a cross-sectional study of 157 overweight and obese youth, where physical activity intensity was measured using accelerometers, only vigorous physical activity was consistently associated with a reduced BMI *z* score and improved systolic BP [13]. A 6-month high intensity aerobic training program in obese adolescents was associated with greater improvement in abdominal obesity and cardiovascular health than was a low intensity program [39]. The REACH intervention compared moderate intensity, not low intensity, to high intensity aerobic exercise. It is possible that the additional anthropometric and cardiovascular benefits observed by others are due to the nature of the high intensity activity, that is, interval training rather than sustained aerobic activity.

A four-month group-based behavioral, physical activity and nutrition intervention was associated with a decrease in BMI *z*-score of −0.19, maintained at 24 months, similar to our finding of −0.17 at 12 months [40]. Ho et al. [41] analyzed the effects of 33 lifestyle interventions on cardiometabolic outcomes in overweight children and concluded that interventions generally produced significant weight loss with a mean decrease in BMI of −1.25 kg/m^2^ and BMI *z*-score of −0.10 compared with untreated controls. There were also improvements in fasting insulin, LDL cholesterol, triglycerides, and BP but no differences in HDL cholesterol. This differs from our findings of an improvement in HDL cholesterol but no change in LDL cholesterol, triglycerides, or blood pressure. The improvement in HDL cholesterol with the REACH intervention may be a result of the activity component, which was frequent and maintained for one year. The absence of improvements in LDL cholesterol, triglycerides, and BP may be because these values were in the normal range at study start, conferring reduced metabolic risk compared to some other studies with subjects selected with preexisting insulin resistance, impaired fasting glucose, or impaired glucose tolerance [13, 19]. In view of the significant CVD risk of low HDL cholesterol levels, the improvement in HDL cholesterol observed in the current study provides encouraging evidence that this important outcome can be positively influenced among at-risk obese youth.

Associations between degree of BMI *z*-score change and markers of metabolic and cardiovascular health have been used in an attempt to establish thresholds by which an intervention can be considered clinically effective. Ford et al. [42] defined a decrease in BMI *z*-score of 0.25 as the lowest threshold for achieving clinical significance in metabolic health. However, in a family-based lifestyle intervention, the benefits on health and fitness did not differ according to the degree of BMI *z*-score reduction [43]. The change in BMI *z*-score in the REACH trial was less than 0.25 which may account for the lack of improvement in insulin resistance beyond 6 months. We found an improvement in leptin levels, which was the only adipokine correlated with insulin resistance in a recent study evaluating the adipocytokine profile of obese adolescents [44].

There is evidence to support the efficacy of family involvement in pediatric obesity interventions. The review by Ho et al. [41] demonstrated that almost all effective interventions reported including a family component. Vannucci and Wilfley [45] also concluded that family-based behavioral interventions have consistently demonstrated efficacy in reducing adiposity and CVD risk factors. All potential participants for the REACH trial were screened for family involvement and only deemed eligible if at least one parent or caregiver committed to the study.

Most studies evaluating the impact of metformin therapy on obesity and metabolic and CVD risk are limited by the absence of a comprehensive, intensive lifestyle component [16–19, 21]. A six-month metformin and lifestyle intervention in obese insulin resistant adolescents demonstrated an improvement in insulin resistance but no change in BMI [46]. However the lifestyle intervention in that study was not intense or comprehensive, described as a low-threshold service, and the metformin dose was relatively low at 1,000 mg daily. The BMI *z*-score decreased by 0.10 in the six-month MOCA (Metformin in Obese Children and Adolescents) trial, with a metformin daily dose of 1,500 mg and no lifestyle intervention [19], whereas in our study with a daily MXR dose of 2,000 mg the BMI *z*-score decreased by 0.14 at six months. The authors of the MOCA trial argue that a short course of metformin is safe and may halt further increase in BMI. Our results demonstrate that combining metformin with a lifestyle intervention is more effective with respect to a decrease in BMI *z*-score and sustained at 12 months.

Most evidence supports lifestyle and pharmacological interventions in obese adolescents as having a positive impact on BMI. A recent systematic review of metformin in treating obesity in children that encompassed 14 randomized clinical trials, 12 of which included a lifestyle component, concluded that metformin provides a modest reduction in BMI of 1.38 kg/m^2^ [47]. The components and intensity of the lifestyle interventions in these studies varied significantly. The REACH lifestyle intervention was both comprehensive and intense. Strengths of this study are the components of the lifestyle intervention including the multidisciplinary approach with physical activity, nutritional and behavioral interventions, the high frequency of sessions, and family-centred delivery. The lifestyle intervention was designed to promote translation of healthy lifestyle habits to long term daily life. Another strength of the study was the metformin formulation as MXR permitting once daily dosing and reduced gastrointestinal side effects.

A major limitation of this study is the high attrition rate limiting any meaningful deductions from results after 12 months. Despite being a single-center study that was designed to be community based with convenient access and social support, we struggled to maintain subjects in the trial. Reasons for this were numerous and included loss of interest in study activities, no desire to exercise, perception that the intervention was not effective, increased extracurricular activities, transportation difficulties, and lack of family support. Some participants were highly motivated but exited the study because they had reached a target BMI or had undertaken independent physical activities and were too busy to attend weekly exercise sessions. This is concerning given the efforts to facilitate lifestyle changes by screening for family commitment prior to study entry. In addition most sessions were located in a central facility later in the day, and group activities were family focused. Of note, the family sessions had the poorest attendance. The high attrition rate after twelve months precludes evaluation of our goal of assessing sustainability of the interventions. However some of those who exited the study appeared to have benefited, some with a decrease in BMI compared to study entry and some with increased independent physical activities.

The observed benefits of the REACH intervention were quite modest considering the multiple components of the lifestyle intervention. There are a number of factors to be considered in designing interventions aiming for more significant anthropometric and metabolic benefits in the obese adolescent population. Further study to determine if there are specific physical and psychosocial characteristics differentiating potential responders from nonresponders would be valuable, as would the assessment of other variables affecting responsiveness. Few published adolescent lifestyle interventions have extended beyond 12 months, which may reflect lack of motivation or commitment on the part of this population. More extensive evaluation of determinants for ongoing commitment would facilitate optimization of resources.

It has been demonstrated that the effect of metformin appears to be more evident in studies of 6-month duration compared to those that continued for a year suggesting a decrease in effectiveness over time [42]. If there is no further benefit to be derived from continuing metformin beyond six months, then the importance of combining metformin with a lifestyle intervention is underscored providing a model that potentially could be translated into improved long-term health routines.

In conclusion, a structured lifestyle intervention in combination with MXR was successful in arresting the rise in BMI *z*-score and percent body fat in obese adolescents, independent of initial exercise intensity. This combination led to a decline in BMI *z*-score and % body fat that was sustained at 12 months, highlighting the benefit of a combined lifestyle and MXR intervention. In the long term, sustained lifestyle modification is clearly preferable to prolonged metformin therapy. We would suggest that an initial 6- to 12-month course of MXR, in combination with a lifestyle intervention, be used to promote improvement in BMI and body composition in obese adolescents. This strategy would potentially foster sustained healthy lifestyle changes after withdrawing MXR.

## Figures and Tables

**Figure 1:**
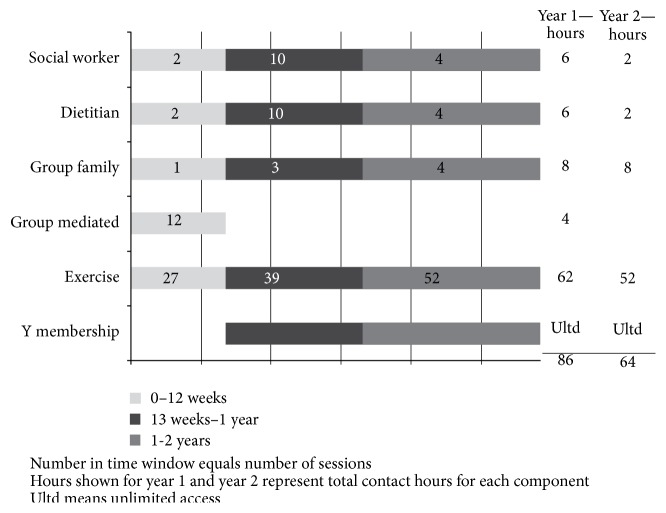
Lifestyle intervention components—frequency and duration.

**Table 1:** Baseline characteristics.

	Metformin		Placebo		*P* value
	Vigorous exercise	Moderate exercise	Vigorous exercise	Moderate exercise	
Age (yr)	13.86 (2.38)	13.39 (2.07)	13.27 (2.19)	14.31 (1.99)	0.47
Sex, male *n*	6 (37.50)	10 (58.82)	6 (33.33)	7 (38.89)	0.43
Ethnicity *n*					
Caucasian	13 (81.25)	14 (82.35)	12 (66.67)	14 (77.78)	0.78
Black	0 (0.00)	0 (0.00)	0 (0.00)	2 (11.11)	
Asian	0 (0.00)	1 (5.88)	1 (5.56)	0 (0.00)	
Native	1 (6.25)	1 (5.88)	0 (0.00)	1 (5.56)	
Other	2 (12.50)	1 (5.88)	5 (27.78)	1 (5.56)	
BMI (kg/m^2^)	34.43 (5.67)	31.56 (5.19)	32.15 (6.34)	31.96 (5.08)	0.46
BMI *z*-score	2.31 (0.31)	2.14 (0.41)	2.15 (1.51)	2.10 (0.38)	0.40
Body fat (%)	46.49 (6.18)	45.25 (3.97)	44.91 (5.71)	44.19 (4.51)	0.68
HOMA	3.27 (1.53)	3.20 (2.77)	4.27 (2.44)	3.27 (1.41)	0.39
FBG (mmol/L)	4.91 (0.34)	4.86 (0.35)	4.96 (0.33)	4.68 (0.59)	0.23
2 hr BG (mmol/L)	5.24 (0.54)	5.55 (1.38)	6.02 (1.59)	5.67 (1.20)	0.35
Leptin ng/mL	39.26 (23.00)	31.54 (13.25)	40.64 (2.39)	36.01 (16.00)	0.58
Adiponectin *µ*g/mL	2.85 (1.58)	2.70 (0.94)	2.25 (1.35)	2.11 (1.16)	0.28
HDL cholesterol (mmol/L)	1.14 (0.34)	1.29 (0.26)	1.25 (0.25)	1.27 (0.44)	0.57
LDL cholesterol (mmol/L)	2.34 (0.60)	2.11 (0.68)	2.31 (0.68)	2.26 (0.72)	0.77
Triglycerides (mmol/L)	1.59 (0.87)	0.92 (0.54)	1.33 (0.65)	1.10 (0.64)	0.04
Systolic BP (mmHg)	111.69 (13.47)	116.24 (13.35)	112.06 (10.44)	111.72 (10.83)	0.56
Diastolic BP (mmHg)	66.31 (9.86)	63.82 (8.20)	70.33 (10.98)	64.59 (10.09)	0.20

Data are means (SD) or numbers in each group.

**Table 2:** Changes in BMI and % body fat.

		Exercise condition	Baseline mean (SD)	13 weeks mean (SD)	6 months mean (SD)	12 months mean (SD)	24 months mean (SD)	Tx *P* value	Time *P* value	Tx ∗ time *P* value
BMI	MXR	Vigorous	34.43 (5.67)	32.20 (6.80)	33.23 (5.62)	32.33 (6.45)	33.83 (6.19)	0.71	0.002	0.09
		Moderate	31.56 (5.19)	31.11 (5.44)	30.98 (5.78)	32.44 (5.24)	33.13 (5.21)			
	Placebo	Vigorous	32.15 (6.34)	32.26 (5.64)	31.48 (5.26)	32.26 (5.34)	33.47 (5.93)			
		Moderate	31.96 (5.09)	32.35 (5.62)	32.69 (5.68)	34.65 (6.00)	34.29 (6.74)			

BMI *z*	MXR	Vigorous	2.31 (0.31)	2.26 (0.34)	2.16 (0.41)	1.99 (0.50)	2.02 (0.56)	0.77	<0.0001	0.13
		Moderate	2.14 (0.41)	2.05 (0.46)	2.02 (0.54)	2.10 (0.49)	2.15 (0.44)			
	Placebo	Vigorous	2.15 (0.41)	2.15 (0.36)	2.07 (0.37)	2.12 (0.40)	2.13 (0.42)			
		Moderate	2.10 (0.38)	2.10 (0.41)	2.10 (0.42)	2.22 (0.41)	2.11 (0.46)			

% over BMI	MXR	Vigorous	79.80 (28.29)	42.75 (10.02)	71.62 (30.24)	64.35 (32.77)	55.67 (30.33)	0.74	0.002	0.11
		Moderate	68.69 (27.13)	66.24 (28.01)	63.31 (29.05)	67.61 (27.94)	70.60 (36.14)			
	Placebo	Vigorous	71.79 (32.98)	44.52 (5.35)	65.22 (29.30)	69.03 (33.37)	69.12 (35.15)			
		Moderate	73.62 (26.27)	66.18 (26.73)	65.97 (25.85)	64.43 (23.69)	67.11 (27.61)			

% body fat	MXR	Vigorous	46.49 (6.18)	42.75 (10.02)	42.70 (8.11)	42.98 (6.98)	45.78 (10.41)	0.86	0.002	0.22
		Moderate	45.25 (3.97)	43.58 (5.07)	42.62 (6.70)	43.82 (5.90)	44.32 (5.19)			
	Placebo	Vigorous	44.91 (5.71)	44.52 (5.35)	44.42 (5.78)	45.07 (4.65)	43.66 (5.08)			
		Moderate	44.19 (4.51)	43.47 (4.98)	44.35 (4.77)	44.53 (4.67)	42.34 (4.20)			

**Table 3:** Changes in metabolic and vascular characteristics.

		Exercise condition	Baseline mean (SD)	6 months mean (SD)	12 months mean (SD)	24 months mean (SD)	Tx *P* value	Time *P* value	Tx ∗ time *P* value
HOMA	MXR	Vigorous	3.27 (1.53)	2.46 (1.56)	2.90 (1.51)	3.00 (1.67)	0.33	0.001	0.03
		Moderate	3.20 (2.77)	1.94 (1.22)	1.94 (1.22)	4.96 (2.39)			
	Placebo	Vigorous	4.27 (2.44)	2.63 (1.53)	3.62 (1.83)	5.64 (4.25)			
		Moderate	3.27 (1.41)	4.00 (2.54)	4.02 (2.07)	4.56 (2.05)			

Fasting BG (mmol/L)	MXR	Vigorous	4.91 (0.34)	4.62 (0.39)	4.75 (0.39)	5.08 (0.50)	0.88	<0.0001	0.42
		Moderate	4.86 (0.35)	4.51 (0.32)	4.82 (0.19)	4.97 (0.22)			
	Placebo	Vigorous	4.96 (0.33)	4.56 (0.47)	4.94 (0.35)	5.13 (0.30)			
		Moderate	4.68 (0.59)	4.64 (0.65)	4.90 (0.39)	4.97 (0.38)			

2 hr BG (mmol/L)	MXR	Vigorous	5.24 (0.54)	5.59 (0.98)	5.65 (0.90)	5.10 (0.66)	0.46	0.81	0.36
		Moderate	5.55 (1.38)	5.17 (1.16)	5.55 (0.97)	5.83 (1.28)			
	Placebo	Vigorous	6.02 (1.59)	5.60 (0.98)	5.78 (0.98)	5.94 (1.51)			
		Moderate	5.67 (1.20)	5.79 (1.15)	5.71 (0.95)	5.50 (0.44)			

Leptin ng/mL	MXR	Vigorous	39.26 (23.00)	30.58 (1.69)	28.79 (17.86)	40.34 (21.07)	0.70	0.0003	0.26
		Moderate	31.54 (13.25)	23.41 (12.85)	28.43 (14.52)	37.80 (19.34)			
	Placebo	Vigorous	40.64 (27.39)	30.97 (23.16)	29.80 (14.11)	34.02 (17.87)			
		Moderate	36.01 (16.00)	35.15 (12.81)	30.94 (11.30)	43.95 (22.37)			

Adiponectin *µ*g/mL	MXR	Vigorous	2.85 (1.58)	3.14 (1.69)	3.54 (1.52)	4.08 (0.82)	0.06	0.62	0.68
		Moderate	2.70 (0.94)	2.67 (0.91)	2.67 (0.83)	2.64 (1.29)			
	Placebo	Vigorous	2.25 (1.35)	2.41 (1.26)	2.40 (1.21)	2.30 (1.15)			
		Moderate	2.11 (1.16)	2.09 (1.23)	2.04 (1.06)	2.22 (1.11)			

Adiponectin leptin ratio	MXR	Vigorous	0.11 (0.11)	0.15 (0.13)	0.19 (0.18)	0.14 (0.11)	0.04	0.05	0.04
		Moderate	0.11 (0.09)	0.17 (0.15)	0.12 (0.07)	0.09 (0.07)			
	Placebo	Vigorous	0.07 (0.07)	0.10 (0.06)	0.09 (0.05)	0.09 (0.09)			
		Moderate	0.06 (0.04)	0.06 (0.04)	0.07 (0.05)	0.06 (0.03)			

HDL cholesterol (mmol/L)	MXR	Vigorous	1.14 (0.39)	1.18 (0.31)	1.25 (0.33)	1.46 (0.39)	0.72	0.002	0.24
		Moderate	1.29 (0.26)	1.24 (0.33)	1.35 (0.39)	1.53 (0.42)			
	Placebo	Vigorous	1.25 (0.25)	1.17 (0.24)	1.23 (0.17)	1.22 (0.17)			
		Moderate	1.27 (0.44)	1.32 (0.41)	1.17 (0.27)	1.29 (0.41)			

LDL cholesterol (mmol/L)	MXR	Vigorous	2.34 (0.60)	2.35 (0.71)	2.22 (0.47)	1.93 (0.47)	0.54	0.48	0.71
		Moderate	2.11 (0.68)	2.01 (0.58)	2.19 (0.49)	2.46 (0.55)			
	Placebo	Vigorous	2.31 (0.68)	2.13 (0.57)	2.36 (0.44)	2.35 (0.86)			
		Moderate	2.26 (0.73)	2.22 (0.69)	2.10 (0.74)	2.13 (0.51)			

Triglycerides	MXR	Vigorous	1.59 (0.87)	1.52 (1.10)	1.19 (0.63)	1.10 (0.71)	0.11	0.10	0.28
		Moderate	0.92 (0.54)	0.91 (0.51)	0.89 (0.46)	0.73 (0.43)			
	Placebo	Vigorous	1.33 (0.65)	1.30 (0.70)	1.12 (0.45)	1.24 (0.82)			
		Moderate	1.10 (0.65)	0.95 (0.58)	1.03 (0.49)	1.13 (0.59)			

Systolic BP *z* score	MXR	Vigorous	−0.02 (1.18)	−0.25 (1.05)	−0.41 (1.11)	0.11 (0.12)			
		Moderate	0.52 (1.16)	0.16 (0.85)	0.11 (0.78)	0.06 (0.76)	0.39	0.23	0.88
	Placebo	Vigorous	0.21 (0.95)	−0.02 (0.86)	0.03 (1.03)	0.18 (0.87)			
		Moderate	0.15 (0.90)	0.20 (1.05)	0.24 (0.92)	0.39 (0.75)			

Diastolic BP *z* score	MXR	Vigorous	0.14 (−0.22)	−0.22 (1.00)	−0.24 (0.81)	−0.04 (0.29)			
		Moderate	−0.08 (0.76)	−0.20 (0.69)	−0.16 (0.65)	−0.47 (0.83)	0.07	0.25	0.80
	Placebo	Vigorous	0.56 (1.06)	0.40 (0.65)	0.32 (0.66)	0.16 (1.00)			
		Moderate	−0.02 (0.86)	0.19 (0.97)	−0.28 (0.64)	0.10 (0.87)			

PAT	MXR	Vigorous	1.68 (0.45)	1.73 (0.41)	1.44 (0.28)	1.76 (0.46)	0.11	0.001	0.80
		Moderate	1.91 (0.93)	1.82 (0.45)	1.65 (0.40)	1.96 (0.50)			
	Placebo	Vigorous	1.70 (0.43)	1.64 (0.42)	1.56 (0.41)	1.46 (0.29)			
		Moderate	1.94 (0.44)	1.92 (0.55)	1.75 (0.57)	1.86 (0.57)			

**Table 4:** Changes in BMI and % body fat for MXR and placebo groups combined.

	Treatment	Baseline mean (SD)	6 months mean (SD)	12 months mean (SD)	Tx *P* value	Time *P* value	Tx ∗ time *P* value
BMI	MXR	32.95 (5.54)	32.07 (5.72)	32.39 (5.65)	0.98	0.04	0.01
	Placebo	32.06 (5.67)	32.04 (5.40)	33.36 (5.66)			

BMI *z*	MXR	2.22 (0.37)	2.08 (0.48)	2.05 (0.49)	0.95	0.001	0.01
	Placebo	2.12 (0.39)	2.08 (0.38)	2.17 (0.40)			

% over BMI	MXR	74.08 (27.84)	67.33 (29.44)	66.19 (29.46)	0.85	0.0004	0.02
	Placebo	68.11 (28.55)	65.57 (27.27)	71.13 (29.77)			

% body fat	MXR	45.81 (5.03)	42.65 (7.13)	43.47 (6.17)	0.75	<0.0001	0.01
	Placebo	44.57 (5.11)	44.39 (5.23)	44.80 (4.56)			

## Abbreviations

MXR: Metformin extended release
BMI: Body mass index
DEXA: Dual X-ray absorptiometry
HOMA-IR: Homeostasis model assessment insulin resistance index
T2D: Type 2 diabetes
CVD: Cardiovascular disease
FBG: Fasting blood glucose
BP: Blood pressure
PAT: Peripheral arterial tonometry.
